# Perceptions and stigma of anti-obesity pharmacotherapy in adult Polish women

**DOI:** 10.3389/fpubh.2025.1687120

**Published:** 2026-01-12

**Authors:** Tomasz Witaszek, Karolina Kłoda, Agnieszka Mastalerz-Migas, Mateusz Babicki

**Affiliations:** 1 Tomasz Witaszek - Indywidualna Praktyka Lekarska, Kraków, Poland; 2 MEDFIT Karolina Kłoda, Szczecin, Poland; 3 Department of Family Medicine, Faculty of Medicine, Wroclaw Medical University, Wrocław, Poland

**Keywords:** anti-obesity medications, BMI, obesity, stigma, women health

## Abstract

**Background:**

Obesity has significant social implications, as individuals often face stigma, negative attitudes, prejudice, and social discrimination. This stigma not only leads to negative psychosocial outcomes but also deters people living with obesity from adopting healthy lifestyles and seeking healthcare services, ultimately compromising their overall health and wellbeing. The primary aim of this study was to assess attitudes and stigma related to anti-obesity pharmacotherapy (AOMs) among adult Polish women. A secondary aim was to evaluate whether age, BMI, and a history of AOM use were associated with these outcomes.

**Methods:**

In this cross-sectional study, 1,043 adult women residing in Poland completed a computer-assisted web interview (CAWI) using a proprietary questionnaire adapted from established stigma scales. Participants were recruited via social media groups focused on lifestyle change and healthcare. Associations with age, BMI, and AOM use history were analyzed using non-parametric tests and Spearman correlations.

**Results:**

Older age was associated with more stigmatizing attitudes toward obesity and AOM use, while younger age was linked to more favorable views. Higher BMI was associated with greater self-stigma among AOM users. A positive history of AOM use was consistently associated with lower stigma, with the lowest levels observed among current users.

**Conclusions:**

Personal experience with AOM use is associated with more favorable attitudes toward pharmacotherapy and lower self-stigma, whereas younger age among AOM users is linked to greater experienced stigma. These findings highlight the importance of public education that frames obesity as a chronic disease and clarifies the evidence-based role of AOMs, particularly for women without personal experience of AOM use.

## 1 Introduction

Obesity is a critical public health problem, with prevalence rates steadily increasing over time. The World Obesity Federation projects that by 2035, 1.9 billion people worldwide will be living with obesity (1). In Poland, approximately 66% of adults have excess body weight, including an estimated 9 million living with obesity (2). Elevated body mass index (BMI) is associated with numerous complications, particularly insulin resistance and type 2 diabetes, arterial hypertension, elevated triglycerides, low HDL cholesterol, and cardiovascular disease (3). Additionally, obesity has significant social implications, as individuals often face stigma, negative attitudes, prejudice, and social discrimination in various settings, including schools, workplaces, and healthcare facilities. This stigma not only leads to negative psychosocial outcomes but also deters persons living with obesity from adopting healthy lifestyles and seeking healthcare services, ultimately compromising their overall health and wellbeing (4).

The etiology of obesity is complex, requiring a multifaceted approach to prevention and treatment at both the population and individual levels (5). Due to the substantial strain obesity puts on national healthcare systems, the development of new therapeutic interventions has become a major focus of research. In recent years, this has led to the emergence of novel treatment options, particularly new incretin-based medications.

Although anti-obesity medications (AOMs) are not a novel class of medication, recent advancements in active substances registered for obesity treatment have significantly improved the efficacy of pharmacological interventions for excess body weight (6). Semaglutide, a GLP-1 analog, and tirzepatide, a dual GLP-1/GIP agonist, are examples of once-weekly injectable medications that have demonstrated considerable therapeutic potential (7). Originally approved for the treatment of type 2 diabetes mellitus, their effects extend beyond weight reduction and appetite suppression. In the recent SELECT study, semaglutide demonstrated a reduction in major adverse cardiovascular events (cardiovascular death, non-fatal myocardial infarction and non-fatal stroke) by more than 20% (8). Tirzepatide has also shown efficacy in the treatment of obstructive sleep apnea, as demonstrated in the SURMOUNT-OSA trials (9).

AOMs have historically been subject to stigmatization, which can be explained in various ways. One of the most prominent causes is societal misconceptions about obesity itself. There is a widespread conception that it results mainly from personal failings, such as hedonism, lack of willpower or laziness, rather than from a complex interplay of genetic, biological, environmental, societal and psychological factors. This (mis)understanding often leads to the opinion that individuals with obesity are unhealthy by choice (10). This stigma extends to the use of pharmacological interventions, with patients often facing judgement for seeking medical treatment instead of relying exclusively on lifestyle changes. Empirical evidence has shown pervasive weight bias surrounding current language used to talk about obesity, and that the use and impact of stigmatizing and harmful language relating to obesity could lead to internalized weight bias, negative emotional responses (eg, disgust), and reduced health-care engagement (11).

Increased mainstream attention on pharmacological treatments has also influenced the perception of AOMs (12). Alongside the portrayal of these drugs in popular media, are concerning reports of their use among celebrities or influencers for aesthetic purposes, rather than as a clinical treatment. These reports also contribute to misconceptions about the required multidisciplinary support that should be offered as part of any clinical treatment programme (11). Additionally, there has been heightened focus on potential side effects, such as acute pancreatitis (13) or suicidal ideations (14), often discussed without adequate context or supporting scientific data. In the past, several pharmacological interventions for obesity were withdrawn from the market due to safety concerns (15), and this historical experience may influence how modern medication is perceived.

To sum up, individuals may hesitate to pursue these medications, fearing criticism, misunderstanding from healthcare providers and peers, and concerns about their own safety. This can lead to delays in effective treatment, feelings of shame, and hinder efforts to address obesity as a chronic disease that often requires a multidisciplinary approach, including medical, behavioral, and lifestyle interventions. This underlines the importance of education about obesity, its treatment methods, and the need to combat the stigma it's associated with.

The primary aim of this study was to assess attitudes and stigma related to anti-obesity pharmacotherapy (AOMs) among adult Polish women. Specifically, we examined general attitudes toward obesity and the use of AOMs in the full study population, stigma directed toward individuals who use AOMs, and self-stigma among women with current or past AOM use. A secondary aim was to evaluate whether age, BMI, and a history of AOM use were associated with these attitudes and stigma-related outcomes. We hypothesized that individuals with experience using AOMs would demonstrate less stigmatizing attitudes, and that age and BMI would be associated with both stigma and self-stigma.

## 2 Subjects and methods

### 2.1 Participants and recruitment

The data for this study were retrieved using a computer-assisted web interviewing (CAWI) survey, with a proprietary questionnaire created on Google Forms. The survey took place between September 30, 2024, and November 30, 2024, and was distributed via different social media platforms, including Facebook and Instagram. The target groups were adult women who were members of online support communities on social media, including groups focused on lifestyle modification (diet, physical activity, weight management) as well as professional groups uniting healthcare workers (physicians, nurses, dietitians, and other health professionals). These communities were selected to reach individuals engaged in lifestyle discussions and those with professional knowledge relevant to the study topic. Participation was voluntary and recruitment was conducted via links shared within these groups. Participants were fully informed about the study's purpose and methodology and provided informed consent before participating. They had the option to withdraw at any time. The inclusion criteria were being aged 18 or older, residing in Poland, and having internet access. The exclusion criteria were incomplete responses, being male, or not providing consent.

The study adhered to the principles of the Declaration of Helsinki. According to the bioethics committee's evaluation (document reference KBkanc 290/2024), the submitted project does not meet the legal criteria of an experiment under current Polish regulations and therefore does not require formal ethical approval. The study adheres to ethical principles in scientific research and respects the rights of survey participants.

### 2.2 The questionnaire

The questionnaire used in this study was not formally validated. However, several items were adapted from previously validated tools used in stigma-related research, including The Stigma Scale (16). The adaptation process involved modifying language to fit the context of AOMs while preserving the original constructs. Formal psychometric validation was not conducted.

The questionnaire began with the collection of socio-demographic data, including age, gender, height, current weight, and highest recorded weight. Participants were also asked about their employment in healthcare and the presence of any chronic conditions.

The subsequent section aimed to evaluate participants' attitudes toward obesity and its treatment methods, including their perceived safety. This section was accessible to all participants and included statements such as “Obesity is a disease” or “The use of medications for obesity is safe.” Responses were recorded on a 4-point Likert-like scale ranging from “Strongly agree” to “Strongly disagree.” Statements were scored from 1 to 4 or reverse-scored, depending on whether the statement was positive or negative, so that higher scores consistently indicated greater levels of stigmatization.

Subsequent questions assessed whether participants were currently using AOMs or had used them in the past. Participants with a positive history of AOMs use were directed to an additional section of the questionnaire. This section included a series of statements designed to evaluate experiences of stigma related to obesity and its treatment. The final three questions in this section specifically targeted self-stigmatization. These statements were scored using a similar 4-point Likert-like scale as in the earlier section, with higher values indicating greater experienced stigma and self-stigma, ensuring consistency in the scoring system.

### 2.3 Statistical analysis

The study involved variables with both qualitative and quantitative attributes. The normality of the distribution was assessed using the Shapiro-Wilk test. Qualitative variable comparisons were performed using the chi-squared test, while for quantitative variables, non-parametric tests such as the Kruskal–Wallis *H* Test or the Mann–Whitney *U* test were employed. The level of correlation between variables was evaluated using the Spearman correlation coefficient (rS). A significance level of 0.05 was set. Statistical analyses were conducted using Statistica 13.0 (TIBCO Software, Palo Alto, CA, United States).

## 3 Results

### 3.1 Characteristics of the study group

We analyzed responses from 1,043 adult female participants residing in Poland, with a mean age of 38.01 ± 8.86 years and a mean BMI of 31.13 ± 8.38 kg/m^2^. In the studied group, 27.1% (*n* = 283) were classified as overweight, 25.2% (*n* = 263) had first-degree obesity, 17.1% (*n* = 178) second-degree obesity, and 10.5% (*n* = 110) third-degree obesity. In total, 20.5% (*n* = 214) identified as healthcare workers. Among all participants, 59.2% reported having chronic diseases, with obesity (62.4%, *n* = 496) and hypothyroidism (43.9%, *n* = 271) being the most prevalent. Additionally, 54% (*n* = 563) reported a positive history of AOM use, and 39.9% (*n* = 416) were currently using these medications at the time of the survey. The sample reflects adult women voluntarily participating through online recruitment channels and was not intended to represent the general population. A detailed presentation of the study group's characteristics is shown in Table 1.

**Table 1:** Characteristics of the study group.

**Variable**		***N* (%)/M ±SD**
Age [years]		38.01 ± 8.86
Height [m]		1.68 ± 0.08
Body Weight [kg]		87.62 ± 20.88
Body Mass Index [kg/m^2^]		31.13 ± 8.38
Healthcare professionals		214 (20.5)
Current pharmacological treatment of obesity		416 (39.9)
Current pharmacological treatment of obesity (*n* = 416)	Tirzepatide	194 (46.6)
	Semaglutide	120 (28.8)
	Liraglutide	90 (21.6)
	Naltrexone/Bupropion	21 (5)
Previous pharmacological treatment of obesity		147 (14)
Previous pharmacological treatment of obesity (*n* = 147)	Tirzepatide	7 (4.8)
	Semaglutide	81 (55.1)
	Liraglutide	75 (51)
	Naltrexone/Bupropion	20 (13.6)
Chronic diseases		617 (59.2)
Chronic diseases (*n* = 617)	Obesity	385 (62.4)
	Hypertension	164 (26.6)
	Cardiovascular diseases other than hypertension	29 (4.7)
	Hypothyroidism	271 (43.9)
	Diabetes mellitus type 2	55 (8.9)
	Osteoarthritis	42 (6.8)
	Depression	163 (26.4)
	Anxiety	131 (21.2)
	Dyslipidemia	76 (12.3)
	Fatty liver disease	70 (11.2)
	Other	252 (40.8)

M, mean; SD, Standard deviation; N, number of participants, kg/m^2^, kilogram(s) per square meter.

### 3.2 Assessment of attitudes toward obesity and its treatment in the entire study group

Higher age was significantly associated with more stigmatizing attitudes, including lower agreement that obesity is a disease or requires treatment, and greater endorsement of statements suggesting that the use of AOMs is an “easy way out” or a reason for shame. In contrast, higher BMI correlated with less stigmatizing views toward others using AOMs, as indicated by lower agreement with statements describing such individuals as lazy or choosing an easy solution. However, BMI was positively correlated with the belief that AOM use is a reason for shame. No significant association was observed between BMI and views on the justification or safety of pharmacological treatment. A more detailed analysis of these correlations is presented in Table 2.

**Table 2:** The correlation between BMI, age, and attitudes toward obesity and its treatment in the entire study group.

**Statement**	**Variable**			
	**Age**		**BMI**	
	***r***	***p***	***r***	***p***
Obesity is a disease.	0.087	**0.005**	0.021	0.499
Obesity requires treatment.	0.084	**0.007**	−0.008	0.804
The use of medications for obesity can be justified.	0.075	**0.016**	−0.039	0.204
The use of medications for obesity is safe.	0.068	0.028	0.023	0.459
People who use medications for obesity are lazy.	0.049	0.109	−0.077	**0.013**
The use of medications for obesity is an “easy way out.”	0.089	**0.004**	−0.112	**< 0.001**
The use of medications for obesity is a reason for shame.	0.099	**0.001**	0.112	**< 0.001**

BMI, body mass index. Significant effects (*p* < 0.05) are marked in bold.

When assessing attitudes in relation to AOMs use, a positive history of AOMs use was, in nearly all instances, associated with reduced stigma. A more detailed overview of this analysis is provided in Table 3.

**Table 3:** Relationship between attitudes toward obesity and its treatment according to the presence or absence of pharmacotherapy history (vs. both previous and current users considered together).

**Statement**	**Pharmacotherapy history status**		***p***
	**Negative**	**Positive**	
	**N/M** ±**SD**	**N/M** ±**SD**	
Obesity is a disease.	1.37 ± 0.63	1.19 ± 0.45	**< 0.001**
Obesity requires treatment.	1.24 ± 0.53	1.13 ± 0.40	**0.020**
The use of medications for obesity can be justified.	1.41 ± 0.65	1.14 ± 0.36	**< 0.001**
The use of medications for obesity is safe.	2.04 ± 0.65	1.77 ± 0.58	**< 0.001**
People who use medications for obesity are lazy.	1.62 ± 0.75	1.35 ± 0.61	**< 0.001**
The use of medications for obesity is an “easy way out.”	1.73 ± 0.82	1.41 ± 0.71	**< 0.001**
The use of medications for obesity is a reason for shame.	1.38 ± 0.74	1.48 ± 0.82	0.096

M, mean; SD, Standard deviation; N, number; kg/m^2^, kilogram(s) per square meter. Significant effects (*p* < 0.05) are marked in bold.

When further distinguishing participants with a positive history of AOMs use into those who used them in the past and those who currently use them, current use demonstrated an even stronger association with less stigmatizing attitudes. Those who discontinued AOMs reported lower levels of stigmatization compared to individuals who had never used the medication. The full analysis is presented in Table 4.

**Table 4:** Relationship between attitudes toward obesity and its treatment by pharmacotherapy history (presence vs. absence), compared with analyses treating previous and current users as separate groups.

**Statement**	**Pharmacotherapy status**			***p***
	**Negative**	**Positive**		
		**Past**	**Current**	
	**M** ±**SD**	**M** ±**SD**	**M** ±**SD**	
Obesity is a disease.	1.37 ± 0.63	1.27 ± 0.53	1.17 ± 0.42	**< 0.001**
Obesity requires treatment.	1.24 ± 0.53	1.17 ± 0.41	1.12 ± 0.39	**< 0.001**
The use of medications for obesity can be justified.	1.41 ± 0.65	1.23 ± 0.45	1.11 ± 0.31	**< 0.001**
The use of medications for obesity is safe.	2.04 ± 0.65	1.91 ± 0.65	1.72 ± 0.55	**< 0.001**
People who use medications for obesity are lazy.	1.62 ± 0.75	1.42 ± 0.65	1.33 ± 0.60	**< 0.001**
The use of medications for obesity is an “easy way out.”	1.73 ± 0.82	1.46 ± 0.72	1.39 ± 0.70	**< 0.001**
The use of medications for obesity is a reason for shame.	1.38 ± 0.74	1.35 ± 0.67	1.53 ± 0.86	**0.012**

M, mean; SD, Standard deviation. Significant effects (*p* < 0.05) are marked in bold.

### 3.3 Assessment of experiences of stigma related to obesity and its treatment among participants with AOMs use history

Analysis of the second part of the questionnaire, showed that age was negatively correlated with both experienced stigma and self-stigma. Although the correlation with BMI was less pronounced, there was a statistically significant positive correlation between calculated BMI and all three self-stigma questions. A more detailed overview is presented in Table 5.

**Table 5:** The correlation between BMI, age, and experiences of stigma related to obesity and its treatment among all users (current and past).

**Statement**	**Variable**			
	**Age**		**BMI**	
	***r***	***p***	***r***	***p***
Society understands the problems of people treated for obesity.	−0.089	**0.035**	0.106	**0.012**
My immediate family knows that I use medications for obesity.	−0.118	**0.005**	0.037	0.375
My friends and acquaintances know that I use medications for obesity.	−0.047	0.268	0.097	**0.021**
I am afraid of my immediate family's reaction to the news that I use medications for obesity.	−0.109	**0.010**	−0.033	0.432
I am afraid of my friends‘ and acquaintances' reaction to the news that I use medications for obesity.	−0.096	**0.022**	0.031	0.457
I am ashamed that I use medications for obesity.	−0.147	**< 0.001**	0.079	0.061
I am afraid of healthcare workers' reaction to the news that I use medications for obesity.	−0.151	**< 0.001**	−0.049	0.249
I feel the need to hide that I use medications for obesity.	−0.132	**0.002**	−0.010	0.809
I have been shamed for using medications for obesity.	−0.083	**0.049**	0.032	0.448
I have been discriminated against because of using medications for obesity.	−0.073	0.084	0.087	**0.039**
When I hear others talking about medications for obesity, I feel bad about myself.	−0.123	**0.003**	0.063	0.136
I consider using medications for obesity to be my personal failure.	−0.128	**0.002**	0.166	**< 0.001**
Because of using medications for obesity, I do not feel good enough.	−0.146	**0.001**	0.146	**0.001**
I feel guilty about using medications for obesity.	−0.108	**0.011**	0.158	**< 0.001**

BMI, body mass index. Significant effects (*p* < 0.05) are marked in bold.

When assessing experienced stigma, individuals currently using medication were more likely to disclose their use to immediate family members. Persons currently using AOMs also demonstrated a strong, statistically significant relationship with self-stigma, with lower self-stigma scores observed across all three questions. Detailed results are presented in Table 6.

**Table 6:** Relationship between experiences of obesity-related stigma and treatment, and the status of pharmacotherapy use (past vs. current).

**Statement**	**Pharmacotherapy status**		***p***
	**Past**	**Current**	
	**M** ±**SD**	**M** ±**SD**	
Society understands the problems of people treated for obesity.	3.56 ± 0.63	3.48 ± 0.67	0.252
My immediate family knows that I use medications for obesity.	2.29 ± 1.45	1.92 ± 1.08	**< 0.001**
My friends and acquaintances know that I use medications for obesity.	2.71 ± 1.16	2.51 ± 1.78	0.077
I am afraid of my immediate family's reaction to the news that I use medications for obesity.	2.06 ± 1.06	2.04 ± 1.44	0.734
I am afraid of my friends‘ and acquaintances' reaction to the news that I use medications for obesity.	2.25 ± 1.08	2.32 ± 1.17	0.594
I am ashamed that I use medications for obesity.	2.20 ± 1.05	2.06 ± 1.05	0.173
I am afraid of healthcare workers' reaction to the news that I use medications for obesity.	2.33 ± 1.10	2.24 ± 1.09	0.422
I feel the need to hide that I use medications for obesity.	2.37 ± 1.13	2.44 ± 1.06	0.469
I have been shamed for using medications for obesity.	1.93 ± 1.05	1.78 ± 0.98	0.166
I have been discriminated against because of using medications for obesity.	1.67 ± 0.96	1.55 ± 0.90	0.190
When I hear others talking about medications for obesity, I feel bad about myself.	2.10 ± 1.04	1.97 ± 1.07	0.200
I consider using medications for obesity to be my personal failure.	2.16 ± 1.08	1.77 ± 0.94	**< 0.001**
Because of using medications for obesity, I do not feel good enough.	1.95 ± 1.04	1.69 ± 0.91	**0.017**
I feel guilty about using medications for obesity.	1.91 ± 1.00	1.66 ± 0.93	**0.008**

M, mean; SD, Standard deviation. Significant effects (*p* < 0.05) are marked in bold.

## 4 Discussion

This study examined general attitudes toward obesity and AOMs, stigma directed toward individuals using AOMs, and self-stigma among women with current or past AOM use. In line with our primary and secondary aims, we also evaluated whether these attitudes were associated with age, BMI, and a history of AOM use. We anticipated that the attitudes toward that method of treatment may depend on age and BMI.

As many as 97% of participants (with 76% strongly agreeing) recognized obesity as a disease, and nearly 98% agreed that it requires treatment. In a study by Swider et al., similar rates were observed, with 94% of respondents recognizing obesity as a disease. These figures are significantly higher than those reported in a 2019 study, where 68% of people living with obesity agreed with that statement (17). Several factors may account for these differences. Participants in the current study were recruited from online lifestyle support communities and professional healthcare groups. As a result, the sample included a relatively high proportion of individuals with an interest in health related topics, and 20% of participants were healthcare professionals. This may limit the generalizability of the findings, as these individuals may demonstrate greater baseline knowledge of the physiological, genetic, and environmental determinants of obesity compared to the general population. Notably, the recognition of obesity as a disease was higher among healthcare professionals, with nearly 98.6% in this study and 88% in prior studies agreeing with this perspective (17, 18). Additionally, increased public and clinical visibility of AOMs may influence how obesity is perceived. However, this relationship was not directly assessed in the present study. Between 2019 and 2023, total prescriptions for semaglutide in the U.S. increased nearly sevenfold (19). In Poland, between 2020 and 2024, almost 700,000 patients received prescriptions for GLP-1 analogs (20). Public health campaigns emphasizing the need for multidisciplinary treatment of obesity as a disease likely further shape these evolving perceptions (21).

Less stigmatizing attitudes toward obesity were positively correlated with younger age. This trend could be influenced by the growing popularity of the body positivity (22) and body neutrality (23) movements, mostly presented on the internet, which challenge traditional stigmas. This cultural shift, while contributing to reducing the stigma associated with obesity and its treatments, also reveals a generational division in perceptions of body image in general (24). Although younger individuals are often reported to place greater emphasis on physical appearance rather than viewing obesity as a disease (25), our study suggests that younger participants in this sample also demonstrated greater acceptance of novel treatment options such as AOMs. This may be a reflection of broader societal attitudes, where younger people are more likely to accept new ideas and try out new solutions (26).

As hypothesized, BMI was an important factor. Individuals who had higher BMI displayed less stigmatizing attitudes toward obesity and its treatment methods. This may be attributed to their firsthand experiences with the limitations of lifestyle interventions (27) and the challenges of managing hunger and satiety often associated with excessive body weight (28). Similarly, a history of AOM use was associated with less stigmatizing attitudes toward pharmacotherapy. Both past and current users were less likely to view patients undergoing treatment as “lazy” or to perceive the use of medication as “an easy way out.” This perspective may result from their understanding and experience that, even with medication, achieving weight loss requires significant effort and lifestyle modifications—a combination that has been demonstrated to be the most effective approach (29).

Interestingly, age was negatively correlated with expressing stigma revolving around AOMs use. Older participants were more likely to disclose their use of medication to immediate family and friends and exhibited fewer self-stigmatizing attitudes. This may stem from their preference for advice from healthcare providers over less reliable sources like social media or online forums. Supporting this notion, a study by Colangeli et al., conducted on an older population with a mean age of 49.1 years, found that 57.7% of participants identified their healthcare provider as their primary source of information about AOMs (30). Additionally, average self-esteem levels tend to increase throughout life, peaking around age 60, which may explain why older participants are less prone to self-stigmatization (31).

Stigmatization can have a substantial impact on the health of individuals living with obesity. Higher BMI levels have been linked to greater delays in seeking and avoiding healthcare services (32). Additionally, overt weight stigma has been significantly associated with poorer treatment outcomes in a 14-week behavioral weight loss program (33).

Safety concerns remain a significant barrier to the broader acceptance of AOMs. Notably, safety was the area where the highest level of concern was expressed across all groups, with only 24% of participants stating they “strongly agree” that these medications are safe. Similarly, a study conducted in Saudi Arabia found that 69.3% and 64.5% of participants believed that AOMs increase the risk of pancreatitis and thyroid tumors, respectively (34). While media reports often emphasize potential risks, the actual incidence of severe side effects is low. The most commonly reported issues are mild and self-limiting gastrointestinal symptoms (35, 36). Participants currently using AOMs expressed less concern about safety, likely due to their personal experience with the treatment or as a factor influencing their continued use. They also reported lower self-stigmatization scores compared to those who discontinued use, which may contribute to their adherence to treatment.

The historical context of AOMs is also important in shaping public perception. For instance, lorcaserin was linked to a potentially increased risk of cancer (37), and sibutramine was associated with an elevated risk of cardiovascular events (38). These past challenges have likely contributed to lingering skepticism regarding the safety of this class of medications. Education about the current evidence on safety, the low incidence of serious adverse events, and the strict regulatory monitoring of modern AOMs may help reduce concerns regarding their safety and long-term use.

Socioeconomic context should also be taken into account when interpreting these findings. In Poland AOMs are not reimbursed, meaning patients must cover the full cost of treatment out-of-pocket. While medical follow-ups can be accessed within the public healthcare system, there are no dedicated, government-funded obesity consultations. Patients are typically seen in primary care, endocrinology or metabolic disease out-patient consults, which may limit comprehensive care. These factors likely contribute to treatment discontinuation, especially among individuals with limited financial resources. They may also introduce bias, as those who can afford AOMs and ongoing care could be more likely to continue treatment and potentially hold different attitudes than those who cannot.

The authors are aware of the limitations of this study, including the lack of representativeness of the study group and the inability to reach individuals without internet access or those outside themed support groups. Research has indicated that self-reported BMI, particularly among individuals with obesity and women, tends to underestimate actual BMI (39). The study did not collect information on the onset or duration of obesity, which limits the ability to differentiate stigma experiences across developmental obesity phenotypes. Furthermore, the cross-sectional nature of this study prevents the investigation of causal pathways. Additionally, there was no information available regarding the duration of AOMs use or the reasons for potential discontinuation. To address these issues, future studies should involve representative patient groups and include objective anthropometric measurements. A further limitation is the use of a non-validated, study-specific questionnaire. Although some items were adapted from existing stigma scales, no validated Polish tool captures AOM-specific stigma. Nevertheless, the strength of our study is undoubtedly its originality and novel approach to the above topic. In addition, the data obtained can be used to prepare appropriate steps to reduce the phenomenon of stigma among patients living with obesity.

Reframing obesity as a chronic condition can help reduce stigma and improve understanding of those seeking treatment, especially within younger patient groups. Efforts to improve public confidence in AOMs should focus on transparent communication about their safety profiles, emphasizing current evidence and distinguishing modern medications from their predecessors.

## Data Availability

The original contributions presented in the study are included in the article/supplementary material, further inquiries can be directed to the corresponding author.
